# Distribution of Parasitic Nematodes Associated with Papaya in Major Production Zones of Burkina Faso

**DOI:** 10.2478/jofnem-2025-0059

**Published:** 2025-12-10

**Authors:** Kouroubi Raïssa Laëtitia Coulibaly, Bouma Thio, Moussa Sondo, Souleymane Yeo, Jacob Sanou, Kadidia Koita, Diana Fernandez

**Affiliations:** Département Production Végétale, Centre National de Recherche Scientifique et Technologique (CNRST), Institut de ľEnvironnement et de Recherches Agricoles (INERA), Bobo Dioulasso, Burkina Faso; Institut de Recherche pour le Développement (IRD); PHIM (Plant Health Institute), Univ. Montpellier, IRD, CIRAD, INRAE, Institute Agro, Montpellier 34394, France; Laboratoire Mixte International (LMI) PathoBios, IRD and INERA, Bobo Dioulasso, Burkina Faso; Laboratoire Biosciences, Département de Biologie et Physiologie Végétale, Université Joseph KI-ZERBO, Ouagadougou, Burkina Faso; UPR Physiologie et Pathologie Végétale, Université Félix HOUPHOUËT-BOIGNY, UFR Biosciences, Abidjan 22, Côte ďIvoire; WASCAL/African Center of Excellence in Climate Change, Biodiversity and Sustainable Agriculture, Pôle Scientifique et ďInnovation, Bingerville, Université Félix HOUPHOUËT-BOIGNY (UFHB), Abidjan 01, Côte ďIvoire

**Keywords:** Burkina Faso, *Carica papaya*, climate factors, cropping systems, detection, diagnosis, *Meloidogyne*, molecular identification, morphology, nematodes, papaya, *Rotylenchulus*

## Abstract

Papaya (*Carica papaya* L.) is an increasingly important fruit crop in Burkina Faso; however, its production is constrained by plant-parasitic nematodes, whose diversity and impact remain poorly documented. A survey of papaya orchards across 9 production regions identified 10 nematode genera, with *Rotylenchulus*, *Helicotylenchus*, *Meloidogyne*, *Scutellonema*, and *Pratylenchus* as the most frequent and abundant. Regional patterns indicated that *Meloidogyne*, *Rotylenchulus*, *Helicotylenchus*, and *Scutellonema* were widespread, and the High-Basins region harbored the richest nematode diversity. Papaya monocultures presented significantly higher nematode densities than intercropped systems, while banana and eggplant associations reduced populations. *Meloidogyne* and *Rotylenchulus* populations were influenced by climatic factors, with frequencies positively correlated with temperature and negatively with rainfall and humidity. Molecular characterization confirmed the widespread presence of *Meloidogyne javanica* and identified *Rotylenchulus reniformis* 0type A in papaya orchards. This study provided the first comprehensive description of nematode distribution in papaya systems, offering valuable insights for developing targeted nematode management strategies in Burkina Faso.

Papaya (*Carica papaya* L.), a tropical fruit crop of the *Caricaceae* family, is cultivated extensively in tropical and subtropical regions worldwide. In 2020, papaya was the third most produced tropical fruit crop in the world (Burns et al., 2022). It is the fourth in terms of global exports after mango, pineapple, and avocado (FAO, 2023) and serves as an important source of vitamins and minerals. In recent years, global papaya production has risen significantly, making it an increasingly important agricultural export commodity – especially for developing countries in Asia and Latin America – where it supports the livelihoods of thousands of smallholder farmers (Evans and Ballen, 2012).

Despite its growing economic importance, papaya production is constrained by several biotic stresses, among which plant-parasitic nematodes (PPN) are particularly damaging. Several nematode genera have been reported in association with papaya worldwide (Khan et al., 2007; Perera et al., 2008; Cornejo-Condori et al., 2021; Indarti and Taryono, 2023). The most widespread and economically damaging nematodes in papaya fields are species of the genera *Meloidogyne* and *Rotylenchulus* (El-Borai and Duncan, 2005; Dias-Arieira et al., 2008; Plena et al., 2022), with yield losses estimated between 15% and 20% (Koenning et al., 1999). Indeed, *Meloidogyne javanica* may develop specific feeding sites in galls and efficiently reproduce in papaya roots (Coulibaly et al., 2025). Nowadays, molecular identification techniques can assist field surveys to identify the most important PPN species in productive areas (Plena et al., 2022; Brenes-Campos et al., 2025; Wanjala et al., 2025). Molecular identification of PPNs typically relies on polymerase chain reaction (PCR) methods for amplification and sequencing of diagnostic loci, such as the internal transcribed spacer (ITS) region or the 18S small subunit *rRNA* gene (Floyd et al., 2002). In addition, species-specific primers have been developed to support species discrimination, particularly for *Meloidogyne* and *Rotylenchulus* species (Zijlstra et al., 2000; Randig et al., 2002; Van den Berg et al., 2016).

In Burkina Faso, papaya cultivation is expanding, with production reaching 18,939 tons in the 2020/2021 season (DGESS, 2019). The crop is grown under various agroecological conditions and farming systems. However, despite the increasing relevance of papaya in the country, little research is known about the parasitic nematode communities associated with its cultivation.

This study aims to address this knowledge gap by (i) identifying the nematode genera associated with papaya in major production areas of Burkina Faso, (ii) characterizing species within the *Meloidogyne* and *Rotylenchulus* genera, and (iii) assessing the cropping systems and agroecological factors influencing nematode population densities. In this study, we present the results of a comprehensive field survey conducted in 61 papaya orchards, along with the characterization of nematode genera and species using both morphological and molecular tools.

## Materials and Methods

### Sample collection

**Figure 1:**
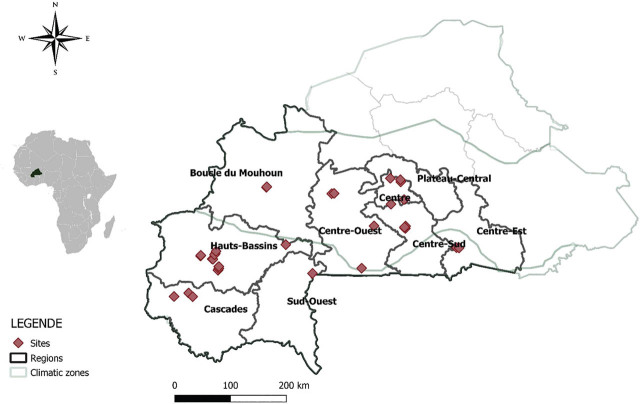
Map of Burkina Faso (administrative map) indicating the sampling sites across nine regions where nematological samples were collected.

The study was conducted in papaya orchards across Burkina Faso during two sampling periods: October 2018 and July 2021. Nine administrative regions representing the main papaya production areas (Figure 1) and comprising 29 localities were surveyed. A total of 61 papaya orchards were surveyed, comprising 26 in 2018 and 45 in 2021. With the exception of seven sites, all orchards had been recently established. The age of papaya trees varied among orchards, ranging from 2 months to 36 months. These regions span the Sudanian and Sudanian-Sahelian agro-climatic zones, which show spatial and temporal variation in rainfall, decreasing along a south-to-north gradient. The Sudanian-Sahelian zone receives < 900 mm of annual rainfall, whereas the Sudanian zone receives between 900 mm and 1,200 mm.

A total of 207 composite soil and root samples were collected from farmers' papaya orchards. Samples were taken from cultivars including ‘Solo’, ‘FBPA1’, and other locally grown but unidentified varieties. A minimum of three samples per orchard were collected. For each sampling site, metadata such as date, GPS coordinates, region, province, locality, cultivar type, cropping history, and intercropping practices were recorded. Samples were transported to the nematology laboratory of Institut de ľEnvironnement et de Recherches Agricoles (INERA) in Farako-Ba for morphological analysis of soil and root nematodes.

### Nematode extraction and morphological identification

Soil nematodes were extracted using the Seinhorst elutriator method (Seinhorst, 1962), and root nematodes were extracted using the Seinhorst sprinkler method (Seinhorst, 1950). After extraction, nematodes were observed under an inverted microscope (Hund Wilovert, Serial# 202139 (Wetzlar, DE, Germany) and morphologically identified using the taxonomic key of Mai and Lyon (1975). Nematode populations were quantified as number per cubic decimeter (n/dm^3^) of soil or per gram (n/g) of roots.

### Nematode population density assessment

Population density was calculated as the mean number of nematodes in all samples in which a particular genus was present, expressed as n/dm^3^ of soil or n/g of roots.

The frequency of each nematode genus was computed as the proportion of samples in which the genus was present relative to the total number of samples (Boag, 1993). A bipartite diagram representing nematode genera across the regions was generated to visualize the distribution of nematode genera and cultivars in the surveyed areas.

### Frequency, abundance of nematodes, and climatic factors

Monthly climatic data of papaya orchards were retrieved for the 2018–2022 period (covering the survey interval) from NASA's POWER Project using the Nasapower R package (version 4.0.9; Sparks, 2018). Variables included relative humidity at 2 m above ground (RH2M), dew point temperature (T2MDEW), maximum air temperature (T2M_MAX), mean air temperature (T2M), and minimum air temperature (T2M_MIN). These data were extracted based on the geographic coordinates of each sampled orchards (61) across the nine papaya-producing administrative regions. Monthly averages of each climatic variable were correlated with the frequency and abundance of *Meloidogyne* and *Rotylenchulus* to determine the strength of association between climatic variables (averaged >5 years) and nematode infestation severity. All statistical analyses were conducted using R software (version 4.3) (R Core Team, 2021).

### Effect of cropping systems on nematode population densities

To assess the effect of cropping systems on nematode population densities, intercropping patterns (papaya monoculture, papaya–banana, papaya–eggplant) were observed in papaya orchards and documented through a pre-established survey form. Twenty-seven orchards were sampled per cropping system. Biplots and hierarchical clustering analyses were performed. To identify the significant differences of the mean number of nematodes among cropping systems, the Kruskal-Wallis test was followed by post-hoc Dunn's test at a significant level of *P* < 0.05. All statistical analyses were performed using R version 4.3 (R Core Team, 2021).

### DNA extraction and PCR assays

To enable species-level identification of the main nematodes associated with papaya in Burkina Faso, molecular characterization of 138 root or soil samples with *Meloidogyne* and *Rotylenchulus* was conducted at the Institut de Recherche pour le Développement (IRD) in Montpellier, France. Two DNA extraction methods were employed: a modified protocol from Holterman et al. (2006) for root nematodes, where β-mercaptoethanol was replaced by dithiothreitol (DTT), and the Qiagen DNeasy PowerSoil Pro Kit (Qiagen S.A.S., Courtaboeuf, France) for soil nematodes to overcome soil-derived PCR inhibitors (Roose-Amsaleg et al., 2001). Aliquots of 300 nematodes per sample were prepared in 2-ml Eppendorf tubes. One aliquot per sample was used to extract DNA. When necessary (no DNA or weak concentrations), extraction was repeated on additional aliquots. DNA extracted using the Holterman protocol was diluted 1:10, 1:20, and 1:50 in sterile distilled water before PCR amplification to avoid inhibition of PCR assays by soil or root chemicals and to permit repeating multiple PCR assays with different primer pairs. In general, PCR reactions were tested with a dilution of 1:50. Extracted DNA was stored at −20°C for future use.

The DNA was then amplified by PCR using universal primers for nematodes such as Nem-18S (Forward: CGCGAATRGCTCATTACAACAGC, Reverse: GGGCGGTATCTGATCGCC; Floyd et al., 2002) to check for nematode DNA presence. For *Meloidogyne* spp. detection, specific primers such as MIG (Forward: ACACAGGGGAAAGTTTGCCA, Reverse: GAGTAAGGCGAAGCATATCC; Zijlstra et al., 2000) and Mjav (Forward: 5GGTGCGCGATTGAACTGAGC, Reverse: CAGGCCCTTCAGTGGAACTATAC; Randig et al., 2002) were used. To test for *Rotylenchulus reniformis* type A and B presence, the D2A-F primer (ACAAGTACCGTGAGGGAAAGTTG) was tested as a forward primer with the type A reverse primers R1A-R (GAAAAGGCCTACCCAATGTG) or R2A-R (CCCGATACCATTTCCATACAA G) or the type B primer R1B-RCACAGARCCCRAGCAGCCA) as described in Van den Berg et al. (2016). All PCR reactions were performed in a thermocycler (MyCycler™ Thermal Cycler, Bio-Rad, Marnes-la-Coquette, France) in a reaction volume of 25 μl with different characteristics depending on the type of primers. In addition, the sequences of a subset of amplicons from PCR reactions were checked after Sanger sequencing at Genewiz (Steiβlingen, Germany). Amplicons were sequenced that were obtained with Nem primers (five soil samples) *Rotylenchulus* type A (three soil samples), MIG (eight root samples), and Mjav (three root samples). Sequencing was performed bidirectionally. Raw sequences were trimmed, aligned, and manually adjusted, and consensus sequences were generated in Geneious (version 8.1.8 created by Biomatters, available from http://www.geneious.com) (Kearse et al., 2012). The genetic relatedness of sequences obtained with other nematode sequences in the GenBank database was determined using the nucleotide basic local alignment search tool (BLASTN) (https://blast.ncbi.nlm.nih.gov/Blast.cgi) with default parameters (Altschul et al., 1990).

## Results

### Frequency and abundance of parasitic nematodes associated with papaya

Following the survey and sampling across the 9 papaya-producing regions, 10 nematode genera from 7 families were identified based on morphological criteria (Table 1). The most frequently encountered soil nematodes belonged to the genera *Helicotylenchus* (84.5%), *Rotylenchulus* (84.1%), *Meloidogyne* (68.6%), *Scutellonema* (52.7%), and *Pratylenchus* (33.3%). In terms of abundance in soil samples, the highest population densities were recorded for the genera *Rotylenchulus* (93,800 n/dm^3^ of soil), followed by *Helicotylenchus* (9,800 n/dm^3^ of soil), *Meloidogyne* (7,880 n/dm^3^ of soil), *Scutellonema* (3,100 n/dm^3^ of soil), and *Pratylenchus* (2,600 n/dm^3^ of soil). In root samples, *Meloidogyne* was observed with a frequency of 42%, reaching a maximum density of 1,573 n/g of roots.

**Table 1:** Maximum density (n/dm^3^ of soil or n/g of roots) and frequency (%) of parasitic soil and root nematodes associated with papaya in Burkina Faso.

**Family**	**Genus**	**Density**	**Frequency**
Soil nematodes			

Hoplolaimidae	*Rotylenchulus*	93,800	84.1
Hoplolaimidae	*Helicotylenchus*	9,800	84.5
Heteroderidae	*Meloidogyne*	7,880	68.6
Hoplolaimidae	*Scutellonema*	3,100	52.7
Pratylenchidae	*Pratylenchus*	2,600	33.3
Telotylenchidae	*Tylenchorhynchus*	400	14.0
Criconematidae	*Criconemoïdes*	60	12.1
Longidoridae	*Xiphinema*	40	4.3
Trichodoridae	*Trichodorus*	240	2.9
Telotylenchidae	*Telotylenchus*	40	0.48

Root nematodes			

Heteroderidae	*Meloidogyne*	1,573	42.0
Pratylenchidae	*Pratylenchus*	219	13.0
Hoplolaimidae	*Rotylenchulus*	89	10.6
Hoplolaimidae	*Helicotylenchus*	25	8.2
Hoplolaimidae	*Scutellonema*	2	1.0

### Distribution of papaya varieties by region

During the survey, two main papaya varieties were identified in the orchards: Solo, FBPA1, and a third group consisting of unknown varieties. A distribution map of the papaya varieties produced was developed based on the nine surveyed regions, to identify the type and percentage of varieties grown in papaya orchards across Burkina Faso (Table 2).

**Table 2:** Distribution (%) of papaya varieties across nine production regions in Burkina Faso.

Region	Papaya variety		
	Solo	FBPA1	nd.
Boucle du Mouhoun	67	0	33
Centre-South	85	0	17
Cascades	25	62	12
Centre	67	17	17
Plateau Central	67	0	33
Centre-East	75	0	25
South-West	100	0	0
Centre-West	62	0	38
High-Basins	68	26	6

nd, not determined.

The Solo variety was thus produced in all nine surveyed regions, unlike the FBPA1 variety, which was only cultivated in the Centre, High-Basins, and Cascades regions. In addition to the identified varieties, several producers were unaware of the specific varieties grown in their orchards, a trend observed in all surveyed regions except the South-West, where only the Solo variety was produced.

### Distribution of nematodes by region

**Figure 2:**
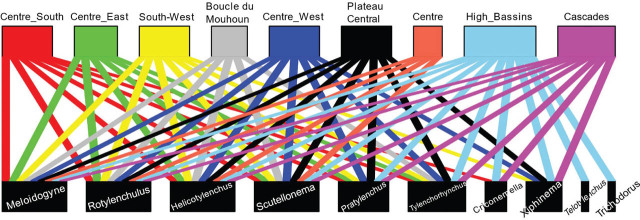
Bipartite diagram illustrating the distribution of nematode genera (down) by region (up) in Burkina Faso. A connecting line indicates the presence of a specific genus in a region. Boxes are sized according to the number of connections established.

The bipartite diagram (Figure 2) reveals that four nematode genera – *Meloidogyne*, *Rotylenchulus*, *Helicotylenchus*, *Scutellonema* – were common to all regions. In contrast, *Telotylenchus* and *Trichodorus* were specific to the High-Basins region. The greatest genus diversity was recorded in the High-Basins region, where all 10 identified genera were present. This was followed by the Cascades region, which harbored eight genera, differing from the High-Basins by the absence of *Telotylenchus* and *Trichodorus*. The lowest genus diversity was observed in the Centre (four genera) and Boucle du Mouhoun (five genera).

### Structure of nematode communities according to papaya-based cropping systems

**Figure 3:**
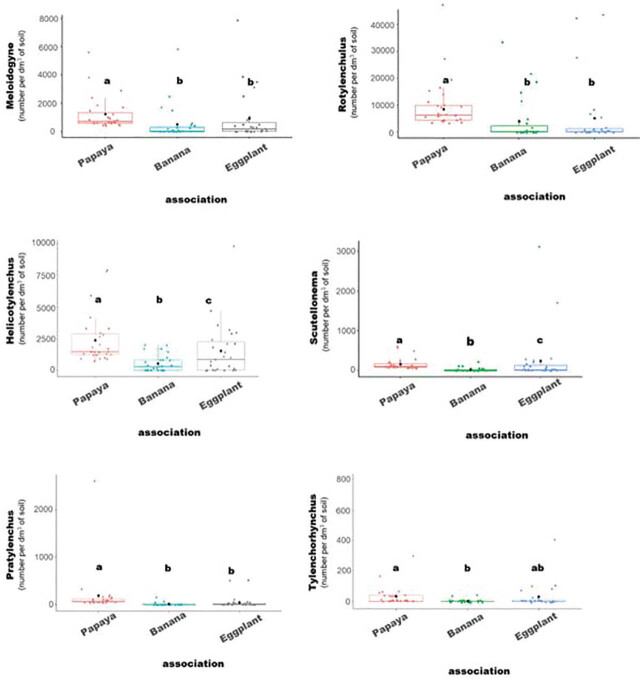
Box plots illustrating nematode population densities (*Meloidogyne* spp., *Rotylenchulus* spp., *Helicotylenchus* spp., *Scutellonema* spp., *Pratylenchus* spp., and *Tylenchorhynchus* spp.) according to the pure papaya cultivation system, papaya–banana associations, and papaya–eggplant associations. The study was conducted during a survey/collection of soil samples from 27 plots in papaya-based cropping systems to compare the impact of each system on nematode population densities. Different letters indicate statistically significant differences between groups when tested with the Kruskal–Wallis test, followed by post-hoc Dunn's test (*P* < 0.05).

Three papaya-based cropping systems were found to influence the population densities of nematodes in papaya orchards: monoculture of papaya, papaya–banana intercropping, and papaya–eggplant intercropping (Table 3; Figure 3). The study demonstrated that the papaya-based cropping systems significantly influence the population densities of parasitic nematodes associated with papaya.

**Table 3:** Parasitic nematodes associated with papaya according to the cropping system (pure or associated to banana or eggplant).

**Nematode**	**Papaya**	**Papaya + Banana**	**Papaya + Eggplant**	**Global Average**	***P*-value**
*Meloidogyne*	1,247 ± 1,205	525 ± 1,226	952 ± 1,798	908	Papaya vs Banana (*P* = 7.17e−06)
					Papaya vs Eggplant (*P* = 0.000758)
*Rotylenchulus*	11,767 ± 17,355	4,176 ± 8,470	5,250 ± 1,2217	7,064	Papaya vs Banana (*P* = 3.81e−05)
					Papaya vs Eggplant (*P* = 2.07e−05)
*Helicotylenchus*	2,391 ± 1,974	546 ± 2,126	1,552 ± 671	1,496	Papaya vs Banana (*P* = 1.22e−06)
					Papaya vs Eggplant (*P* = 0.02)
*Scutellonema*	161 ± 153	24 ± 51	239 ± 659	141	Papaya vs Banana (*P* = 5.26e−08)
					Papaya vs Eggplant (*P* = 0.005)
*Pratylenchus*	184 ± 487	10 ± 33	49 ± 132	81	Papaya vs Banana (*P* = 2.85e−09)
					Papaya vs Eggplant (*P* = 9.61e−07)
*Tylenchorhynchus*	33 ± 64	3 ± 11	30 ± 81	22	Papaya vs Banana (*P* = 0.002)

*Meloidogyne* spp. exhibited an average density of 908 n/dm^3^ of soil, peaking in papaya monocultures (1,247 n/dm^3^ of soil). Papaya–eggplant and papaya–banana intercropping systems recorded lower densities, with highly significant differences between cropping systems. *Rotylenchulus* spp. showed the highest average density among all genera, with 7,064 n/dm^3^ of soil, and a marked predominance in papaya monocultures (11,767 n/dm^3^ of soil). This genus was significantly less abundant in the banana and eggplant intercropping systems. For *Helicotylenchus* spp., the overall average density was 1,496 n/dm^3^ of soil, again peaking in pure papaya orchards (2,391 n/dm^3^ of soil). Intercropped systems showed significantly lower densities. Although less abundant overall, *Scutellonema* spp. showed a clear preference for eggplant (238 n/dm^3^ of soil), with statistically significant differences among all cropping systems. *Pratylenchus* spp. also favored papaya, with an average density of 184 n/dm^3^ of soil, compared to only 49 n/dm^3^ in eggplant and 10 n/dm^3^ in banana systems, differences that were highly significant. Finally, *Tylenchorhynchus* spp. was generally less abundant (22 n/dm^3^ of soil on average), but mainly detected in papaya and eggplant fields, with significantly lower densities in banana associations (3 n/dm^3^ of soil).

Overall, papaya monoculture promoted the highest nematode densities across most genera, highlighting its key role in nematode proliferation. In contrast, intercropping, particularly with banana, appeared to exert a moderating effect on nematode populations.

**Figure 4:**
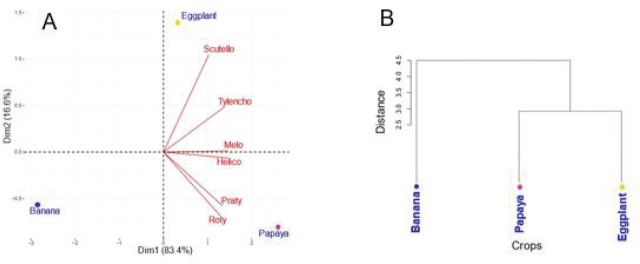
Distribution of parasitic nematodes associated with papaya according to cropping systems (pure or associated with banana or eggplant): (A) Biplot of PPN in interaction with cropping systems. (B) Hierarchical cluster analysis showing similarities between cropping systems based on nematode population densities. PPN, plant-parasitic nematode.

The biplot (Figure 4A) illustrating the distribution of the main papaya-associated nematode genera in relation to papaya-based cropping systems revealed that the first two axes accounted for 83.4% and 16.6% of the total variance, respectively. This plot showed a strong positive correlation between the papaya–eggplant intercropping system and the genera *Scutellonema* spp. and *Tylenchorhynchus* spp. Conversely, papaya monoculture was associated with high population densities of *Rotylenchulus* spp., *Pratylenchus* spp., *Helicotylenchus* spp., and *Meloidogyne* spp.

According to the hierarchical cluster analysis (Figure 4B), the cropping systems involving papaya and eggplant shared similar nematode population structures, while banana intercropping formed a distinct cluster, indicating a different nematode community profile.

### Effect of climatic factors on the distribution of nematodes

#### Frequency and abundance of Meloidogyne spp.

**Figure 5:**
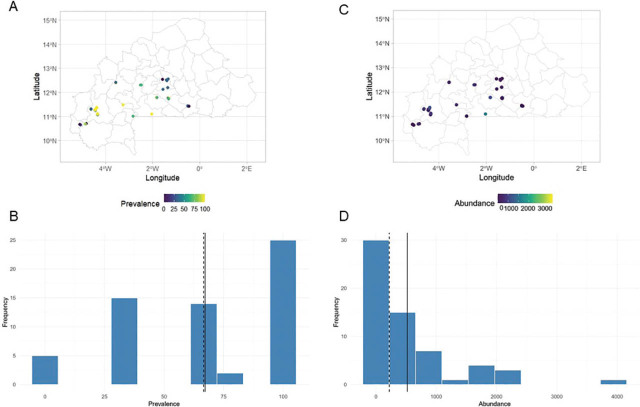
Distribution maps of the prevalence (A,B) and abundance (C,D) of *Meloidogyne* spp. root samples of 61 papaya orchards across Burkina Faso.

An overview of the distribution of *Meloidogyne* spp. in the 61 orchards surveyed is presented in Figure 5. Nematode prevalence (Figures 5A,C) ranged from 0% to 100%, while abundances (Figures 5B,D) ranged from 0 nematodes/dm^3^ to 3,940 nematodes/dm^3^ of soil (Table S1 in Supplementary Materials). The correlation between frequency and abundance of *Meloidogyne* was weak (*r* = 0.474; *P* < 0,001), indicating that the abundance of *Meloidogyne* does not depend on its frequency.

High positive correlations were observed between the average air temperature at 2 m above ground level (T2M), the maximum air temperature at 2 m above ground level (T2M_MAX) and the frequency of *Meloidogyne* spp. with coefficients of 0.75. Conversely, significant negative correlations were observed between precipitation (PREC), relative humidity at 2 meters above ground level (RH2M) and dew point temperature at 2 m above ground level (T2MDEW) with respective coefficients of −0.74, −0.81 and −0.77 (Figure S1A in Supplementary Materials).

The abundance of *Meloidogyne* sp. was strongly positively correlated with RH2M and T2MDEW, with coefficients of 0.63 and 0.70. In contrast, T2M_MIN, T2M_MAX and T2M were negatively correlated with the abundance of *Meloidogyne* spp. in papaya orchards with respective coefficients of −0.84, −0.55 and −0.49 (Figure S1B in Supplementary Materials).

#### Frequency and abundance of Rotylenchulus spp.

**Figure 6:**
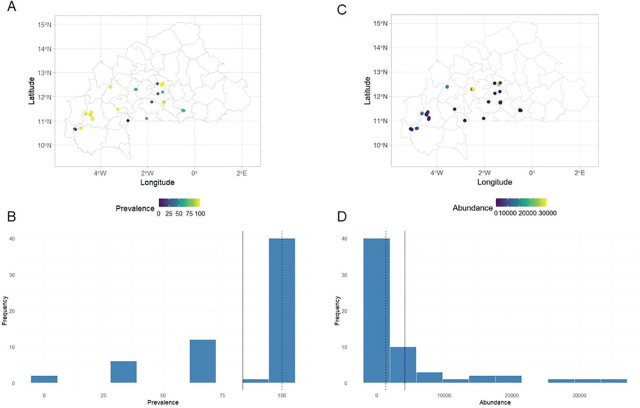
Distribution maps of the frequency (A,B) and abundance (C,D) of *Rotylenchulus* spp. in soil samples of 61 papaya orchards across Burkina Faso.

Figure 6 presents the distribution of *Rotylenchulus* spp. across the 61 surveyed orchards. Nematode prevalence (Figures 6A,C) ranged from 0% to 100% while abundances (Figures 6B,D) varied between 0 n/dm^3^ and 35,100 n/dm^3^ of soil (Table S2 in Supplementary Materials).

Strong positive correlations were found between the average air temperature at 2 m above ground level (T2M), the maximum air temperature at 2 meters above ground level (T2M_MAX), and the frequency of *Rotylenchulus* spp., with respective coefficients of 0.63 and 0.60. In contrast, strong negative correlations were observed between PREC, relative humidity at 2 meters above ground level (RH2M), and the frequency of *Rotylenchulus* spp. with respective coefficients of −0.81 and −0.53 (Figure S2A in Supplementary Materials).

The average air temperature at 2 m above ground level (T2M) and the maximum air temperature at 2 meters above ground level (T2M_MAX) were positively correlated with the abundance of *Rotylenchulus* spp. The coefficients were 0.78 and 0.86, respectively. However, negative correlations were observed between abundance and PREC, relative humidity 2 meters above ground level (RH2M), dew point temperature at 2 m above ground level (T2MDEW), and minimum air temperature at 2 m above ground level (T2M_MIN), with coefficients of −0.80, −0.93, −0.91, and −0.64 (Figure S2B in Supplementary Materials).

#### Molecular characterization of the main parasitic nematodes associated with papaya

Molecular characterization of nematodes was performed on composite samples collected in 2021 from papaya roots or soil associated with papaya to identify the main species of *Meloidogyne* and *Rotylenchulus*. DNA was successfully extracted from 138 samples containing one or both genera, and further amplified by PCR using several primer pairs targeting different nematode genomic regions. Amplified bands were sequenced for a subset (3–8) of randomly chosen isolates to verify their homology with the corresponding nematode species targeted by primers (data not shown). Some amplicons obtained with Nem primers contained mixed sequences from several nematodes.

Using the universal nematode primers Nem-18S, 132 out of the 138 samples produced an amplified fragment of 900 bp, indicating the presence of nematode DNA. Among these 132 samples, 129 tested positive with the Mjav-F/Mjav-R primer pair, specific to *M. javanica* (670 bp amplicon). Additionally, primers specific to the MIG group (*M. incognita*, *M. javanica*, *M. arenaria*) amplified DNA in 114 samples, yielding a 500 bp fragment. Some discrepancies between results of amplification with Mja or MIG primers also occurred, suggesting that other *Meloidogyne* species may be present (for samples MIG+; Mjav−) and that MIG primers do not amplify all *M. javanica* isolates (for samples MIG−; Mjav+).

From 80 soil samples where *Rotylenchulus* had been observed, DNA from *R. reniformis* was amplified in 77 samples using the type A-specific primers (D2AF/R1A-R or D2AF/R2A-R), resulting in amplicons of 142 bp and 320 bp, respectively. No amplification could be observed with the type B primers (D2AF/R1B-R/179 pb), suggesting that only *R. reniformis* type A may be present in papaya orchards surveyed.

## Discussion

This study assessed the diversity and distribution of the major PPNs associated with papaya in nine major production regions of Burkina Faso. Using combined morphological and molecular approaches, 10 nematode genera, belonging to 7 different families were identified. In addition, molecular markers allowed characterization of *M. javanica* as in a large number of samples, and *R. reniformis* (type A) as the *Rotylenchulus* species present in papaya root and soil samples. Other *Meloidogyne* species may be present in some MIG-positive samples since MIG primers can also detect *M. incognita* and *M. arenaria* (Zijlstra et al., 2000). The most frequently encountered genera were *Helicotylenchus*, *Rotylenchulus*, *Meloidogyne*, *Scutellonema*, and *Pratylenchus*. These findings align with reports from other papaya-growing regions such as Latin America and Southeast Asia (Da Silva et al., 2007; Martínez Gallardo et al., 2014; Indarti and Taryono, 2023), suggesting a shared nematode fauna across tropical climates.

*Rotylenchulus reniformis* was the most abundant and widely distributed species, with densities reaching 93,800 n/dm^3^ of soil. Its prevalence confirms its adaptability to diverse environments and its status as a major constraint in tropical horticulture (El-Borai and Duncan, 2005; Martínez Gallardo et al., 2014). *M. javanica*, the second most dominant species, reinforces earlier findings on papaya susceptibility to root-knot nematodes, which are known to cause significant yield losses (Peraza Padilla, 2021). Several other authors have reported the presence of these two species in papaya orchards around the world (Bridge et al., 1996; El-Borai and Duncan, 2005; Dias-Arieira et al., 2008; Perera et al., 2008; Plena et al., 2022; Baldan et al., 2024; Wanjala et al., 2025). According to Peraza Padilla (2021), *M. javanica* is one of the four most commonly identified root-knot nematode species on papaya in Brazil and Colombia, and it was recently reported in Kenya (Wanjala et al., 2025). *M. javanica* is the predominant species of root-knot nematode in regions with well-defined dry seasons (Eisenback et al., 1981), as is the case in Burkina Faso. Molecular confirmation of *M. javanica* emphasizes the importance of integrating molecular diagnostics for accurate species-level identification in nematode surveys.

Differences in nematode community composition across regions are likely influenced by multiple interacting factors, including climate, soil texture, cropping history, and management practices. Regional differences in rainfall (600–1,200 mm), temperature (27–28°C), and humidity may shape nematode population dynamics and spatial patterns (MECV/PANA, 2007). This agrees with previous studies demonstrating the role of edaphic and climatic factors in structuring nematode communities (Norton, 1979; Yeates et al., 1993; Mateille et al., 2014).

Cropping systems significantly affected nematode presence and abundance. Papaya monocultures tended to harbor higher densities of *Meloidogyne* and *Rotylenchulus* compared to intercropped systems, suggesting that continuous cropping of a single host may amplify nematode pressures. Intercropping with banana or eggplant, both known hosts of several PPNs, did not mitigate nematode infestation, possibly due to overlapping host ranges (Netscher and Sikora, 1990; Daneel and De Waele, 2017). Moreover, the predominance of *Scutellonema*, *Pratylenchus*, and *Helicotylenchus* in monocultures suggests that papaya supports a broad spectrum of PPNs, underlining the need for crop rotation or soil health management practices to suppress nematode buildup.

Climatic variables were also key drivers of nematode population dynamics. Relative humidity and dew point were positively correlated with *Meloidogyne* abundance, reflecting its preference for moist conditions conducive to hatching and root penetration. *Rotylenchulus* populations were more responsive to average air temperature and maximum air temperature, consistent with their ability to thrive in warmer climates. These findings support earlier work on climate-nematode interactions (Ankrom et al., 2020; Dutta and Phani, 2023) and suggest that climate change may exacerbate PPN problems in the region.

Overall, the results reveal a rich and diverse PPN community associated with papaya in Burkina Faso, with important implications for disease management. Integrated nematode control strategies – including resistant cultivars, improved cropping systems, and soil health restoration – will be essential for sustainable papaya production in the face of nematode pressure and climatic variability.

## Supplementary Materials

**Table S1:** Prevalence (%) and abundance (nematodes/dm^3^ of soil) of *Meloidogyne* spp. in 61 orchards across 9 regions in Burkina Faso.

**Region**	**Province**	**Locality**	**Latitude**	**Longitude**	**Prevalence Melo**	**Abundance Melo**
B. Mouhoun	Mouhoun	Badara	12.407022	−3.562287	33.33	200
B. Mouhoun	Mouhoun	Sagala1	12.39802	−3.567907	33.33	60
B. Mouhoun	Mouhoun	Sagala2	12.395486	−3.571156	100	920
Cascades	Comoe	Karfiguela	10.69876667	−4.817066667	0	0
Cascades	Comoe	Tengrela	10.68705	−4.8572	83.33	358
Cascades	Leraba	Douna1	10.6449828	−5.052942	33.33	40
Cascades	Leraba	Douna2	10.667974	−5.102256	66.67	30
Cascades	Leraba	Douna3	10.677187	−5.099382	0	0
Centre	Kadiogo	Koubri1	12.202186	−1.353772	33.33	20
Centre	Kadiogo	Koubri2	12.201433	−1.355744	33.33	60
Centre	Kadiogo	Pabre	12.5443	−1.5745	0	0
Centre	Ziniare	Loumbila1	12.487557	−1.407013	33.33	200
Centre	Ziniare	Loumbila2	12.559418	−1.337209	33.33	40
Centre	Ziniare	Noungou	12.521811	−1.41213	33.33	20
Centre-East	Boulgou	Bagre1	11.4302	−0.5124	100	2,240
Centre-East	Boulgou	Bagre2	11.4278	−0.5156	66.67	1,590
Centre-East	Boulgou	Bagre3	11.4519	−0.5143	33.33	20
Centre-East	Boulgou	Bega2	11.4254	−0.4695	0	0
Centre-South	Bazega	Dayassmnoore1	11.750413	−1.349181	66.67	20
Centre-South	Bazega	Dayassmnoore2	11.753409	−1.348793	66.67	30
Centre-South	Bazega	Koupela	11.7595	−1.3469	0	0
Centre-South	Bazega	Lilboure	11.739128	−1.331465	66.67	50
Centre-South	Bazega	SaponeTanghin	12.128827	−1.562903	33.33	40
Centre-South	Bazega	Timboe	11.7755	−1.3287	100	900
Centre-West	Sanguie	Ekoulkoala1	12.297419	−2.517648	100	2,133
Centre-West	Sanguie	Ekoulkoala2	12.300701	−2.516541	66.67	700
Centre-West	Sanguie	Reo	12.300894	−2.479187	66.67	250
Centre-West	Sissili	Zo	11.099	−2.0365	100	1,587
Centre-West	Ziro	Bakata1	11.7786	−1.8372	33.33	20
Centre-West	Ziro	Bakata2	11.7792	−1.8347	100	720
Centre-West	Ziro	Bakata3	11.7812	−1.8273	66.67	950
Hauts-Bassins	Houet	Bama1	11.335399	−4.388538	100	153
Hauts-Bassins	Houet	Bama2	11.366845	−4.380663	100	60
Hauts-Bassins	Houet	Bama3	11.360086	−4.382549	66.67	1,100
Hauts-Bassins	Houet	Bama4	11.36755	−4.379316667	100	147
Hauts-Bassins	Houet	Bama5	11.36035	−4.382866667	100	353
Hauts-Bassins	Houet	Bama6	11.36613333	−4.381	100	1,040
Hauts-Bassins	Houet	Diarradougou1	11.2474	−4.4392	100	320
Hauts-Bassins	Houet	Diarradougou10	11.31128333	−4.412	100	420
Hauts-Bassins	Houet	Diarradougou2	11.2742	−4.4427	100	3,940
Hauts-Bassins	Houet	Diarradougou3	11.3016	−4.429	100	1,660
Hauts-Bassins	Houet	Diarradougou4	11.24376667	−4.428983333	100	493
Hauts-Bassins	Houet	Diarradougou5	11.24835	−4.439516667	66.67	970
Hauts-Bassins	Houet	Diarradougou6	11.25136667	−4.440333333	66.67	100
Hauts-Bassins	Houet	Diarradougou7	11.25511667	−4.444816667	83.33	432
Hauts-Bassins	Houet	Diarradougou8	11.2788	−4.440866667	33.33	20
Hauts-Bassins	Houet	Diarradougou9	11.28433333	−4.440116667	100	27
Hauts-Bassins	Houet	Farako-Ba1	11.095474	−4.332357	100	347
Hauts-Bassins	Houet	Farako-Ba2	11.12648333	−4.333733333	100	423
Hauts-Bassins	Houet	Farako-Ba3	11.12623333	−4.333283333	100	267
Hauts-Bassins	Houet	Finlande	11.12623333	−4.333283333	100	473
Hauts-Bassins	Houet	Flasso	11.12623333	−4.333283333	66.67	220
Hauts-Bassins	Houet	Koumbadougou	11.295473	−4.627477	100	133
Hauts-Bassins	Houet	Lesso	11.067135	−4.345465	66.67	20
Hauts-Bassins	Houet	Soungalodaga1	11.305436	−4.621993	33.33	320
Hauts-Bassins	Houet	Soungalodaga2	11.313764	−4.620531	33.33	20
Hauts-Bassins	Houet	Soungalodaga3	11.313179	−4.618558	33.33	480
Hauts-Bassins	Tuy	Founzan	11.477695	−3.255886	100	333
South-West	Ioba	Ouessa1	11.0167	−2.8261	100	2,247
South-West	Ioba	Ouessa2	11.0142	−2.8269	100	1,907
South-West	Ioba	Ouessa3	11.0094	−2.83	66.67	60

**Table S2:** Prevalence (%) and abundance (nematodes/dm^3^ of soil) of *Rotylenchulus* spp. in 61 orchards across 9 regions in Burkina Faso.

**Region**	**Province**	**Locality**	**Latitude**	**Longitude**	**Prevalence Roty**	**Abundance Roty**
B. Mouhoun	Mouhoun	Badara	12.407022	−3.562287	100	18,433
B. Mouhoun	Mouhoun	Sagala1	12.39802	−3.567907	100	1,600
B. Mouhoun	Mouhoun	Sagala2	12.395486	−3.571156	100	1,567
Cascades	Comoe	Karfiguela	10.69876667	−4.817066667	100	18,927
Cascades	Comoe	Tengrela	10.68705	−4.8572	100	7,497
Cascades	Leraba	Douna1	10.6449828	−5.052942	0	0
Cascades	Leraba	Douna2	10.667974	−5.102256	33.33	20
Cascades	Leraba	Douna3	10.677187	−5.099382	33.33	20
Centre	Kadiogo	Koubri1	12.202186	−1.353772	100	10,353
Centre	Kadiogo	Koubri2	12.201433	−1.355744	66.67	1,610
Centre	Kadiogo	Pabre	12.5443	−1.5745	33.33	20
Centre	Ziniare	Loumbila1	12.487557	−1.407013	100	27,567
Centre	Ziniare	Loumbila2	12.559418	−1.337209	100	5,933
Centre	Ziniare	Noungou	12.521811	−1.41213	100	31,987
Centre-East	Boulgou	Bagre1	11.4302	−0.5124	66.67	70
Centre-East	Boulgou	Bagre2	11.4278	−0.5156	100	20
Centre-East	Boulgou	Bagre3	11.4519	−0.5143	66.67	1,840
Centre-East	Boulgou	Bega2	11.4254	−0.4695	66.67	40
Centre-West	Sanguie	Ekoulkoala1	12.297419	−2.517648	100	15,433
Centre-West	Sanguie	Ekoulkoala2	12.300701	−2.516541	66.67	2,050
Centre-West	Sanguie	Reo	12.300894	−2.479187	66.67	35,100
Centre-West	Sissili	Zo	11.099	−2.0365	66.67	20
Centre-West	Ziro	Bakata1	11.7786	−1.8372	33.33	60
Centre-West	Ziro	Bakata2	11.7792	−1.8347	100	4,413
Centre-West	Ziro	Bakata3	11.7812	−1.8273	33.33	20
Centre-South	Bazega	Dayassmnoore1	11.750413	−1.349181	100	220
Centre-South	Bazega	Dayassmnoore2	11.753409	−1.348793	100	1,847
Centre-South	Bazega	Koupela	11.7595	−1.3469	100	67
Centre-South	Bazega	Lilboure	11.739128	−1.331465	100	747
Centre-South	Bazega	Sapone-Tanghin	12.128827	−1.562903	33.33	160
Centre-South	Bazega	Timboe	11.7755	−1.3287	66.67	40
Hauts-Bassins	Houet	Bama1	11.335399	−4.388538	100	5,200
Hauts-Bassins	Houet	Bama2	11.366845	−4.380663	100	2,093
Hauts-Bassins	Houet	Bama3	11.360086	−4.382549	100	1,847
Hauts-Bassins	Houet	Bama4	11.36755	−4.379316667	100	6,853
Hauts-Bassins	Houet	Bama5	11.36035	−4.382866667	100	773
Hauts-Bassins	Houet	Bama6	11.36613333	−4.381	100	5,213
Hauts-Bassins	Houet	Diarradougou1	11.2474	−4.4392	66.67	20
Hauts-Bassins	Houet	Diarradougou10	11.31128333	−4.412	100	1,473
Hauts-Bassins	Houet	Diarradougou2	11.2742	−4.4427	100	80
Hauts-Bassins	Houet	Diarradougou3	11.3016	−4.429	100	2,613
Hauts-Bassins	Houet	Diarradougou4	11.24376667	−4.428983333	100	640
Hauts-Bassins	Houet	Diarradougou5	11.24835	−4.439516667	88.88	3,723
Hauts-Bassins	Houet	Diarradougou6	11.25136667	−4.440333333	66.67	1,480
Hauts-Bassins	Houet	Diarradougou7	11.25511667	−4.444816667	100	5,533
Hauts-Bassins	Houet	Diarradougou8	11.2788	−4.440866667	100	207
Hauts-Bassins	Houet	Diarradougou9	11.28433333	−4.440116667	100	106
Hauts-Bassins	Houet	Farako-Ba1	11.095474	−4.332357	100	4,493
Hauts-Bassins	Houet	Farako-Ba2	11.12648333	−4.333733333	100	843
Hauts-Bassins	Houet	Farako-Ba3	11.12623333	−4.333283333	100	2,140
Hauts-Bassins	Houet	Finlande	11.12623333	−4.333283333	100	1,367
Hauts-Bassins	Houet	Flasso	11.12623333	−4.333283333	100	340
Hauts-Bassins	Houet	Koumbadougou	11.295473	−4.627477	100	107
Hauts-Bassins	Houet	Lesso	11.067135	−4.345465	100	133
Hauts-Bassins	Houet	Soungalodaga1	11.305436	−4.621993	66.67	180
Hauts-Bassins	Houet	Soungalodaga2	11.313764	−4.620531	100	1,213
Hauts-Bassins	Houet	Soungalodaga3	11.313179	−4.618558	100	15,033
Hauts-Bassins	Tuy	Founzan	11.477695	−3.255886	100	87
South-West	Ioba	Ouessa1	11.0167	−2.8261	100	1,673
South-West	Ioba	Ouessa2	11.0142	−2.8269	66.67	110
South-West	Ioba	Ouessa3	11.0094	−2.83	0	0
